# Thyroid blood flow in inferior thyroid artery as predictor for increase in levothyroxine dosage during pregnancy in women with Hashimoto's thyroiditis - a retrospective study

**DOI:** 10.1186/s12884-019-2389-1

**Published:** 2019-07-05

**Authors:** Masafumi Kurajoh, Akiyo Yamasaki, Toshiki Nagasaki, Yuki Nagata, Shinsuke Yamada, Yasuo Imanishi, Masanori Emoto, Kanae Takahashi, Kouji Yamamoto, Ayumi Shintani, Masaaki Inaba

**Affiliations:** 1 Department of Metabolism, Endocrinology, and Molecular Medicine, Osaka City University Graduate School of Medicine, 1-4-3, Asahi-machi, Abeno-ku, Osaka, 545-8585 Japan; 2 Department of Medical Statistics, Osaka City University Graduate School of Medicine, Osaka, Japan

**Keywords:** ITA-PSV, Hashimoto’s thyroiditis, Pregnancy, Levothyroxine dosage

## Abstract

**Background:**

We examined whether inferior thyroid artery peak systolic velocity (ITA-PSV) predicts an increase in levothyroxine (LT4) dosage in pregnant women with Hashimoto’s thyroiditis.

**Methods:**

Twenty-two women with Hashimoto’s thyroiditis who were planning and later achieved pregnancy or confirmed as pregnant were enrolled in this retrospective longitudinal observational study. ITA-PSV and thyroid volume were measured using ultrasonography. Serum concentrations of free thyroxine (F-T4), free triiodothyronine (F-T3), and thyroid stimulating hormone (TSH) were simultaneously determined. We adjusted LT4 dosage to maintain serum TSH at < 2.5 μIU/mL (1st trimester) and later at < 3 μIU/mL (2nd, 3rd trimester).

**Results:**

Eighteen patients (81.8%) required an increase in LT4 dosage during pregnancy, of whom 7 (31.8%) required an increase ≥50 μg. Multivariable regression analysis showed that TSH (β = 0.507, *p* = 0.008) and ITA-PSV (β = − 0.362, *p* = 0.047), but not thyroid volume, F-T4, or F-T3, were independently associated with increased LT4 dosage. Receiver-operating characteristic analysis for predicting an increase in LT4 ≥ 50 μg/day showed that the area under the curve (0.905) for ITA-PSV with TSH was not significantly increased (*p* = 0.123) as compared to that (0.743) for TSH alone, whereas integrated discrimination improvement was significantly increased (27.9%, *p* = 0.009).

**Conclusions:**

In pregnant patients with Hashimoto’s thyroiditis, ITA-PSV was a significant predictor of increase in LT4 dosage independent of TSH level, while ITA-PSV plus TSH showed significantly improved predictability as compared to TSH alone. These results suggest that ITA-PSV reflects residual thyroid function and is useful for evaluating the need for increased thyroid hormone production in pregnant patients with Hashimoto’s thyroiditis.

## Background

Hypothyroidism during pregnancy, even when subtle, is considered to increase the risk of miscarriage, gestational hypertension, placental abruption, preeclampsia, and postpartum hemorrhage, as well as psychoneurological disorders in the child [1–4]. Therefore, levothyroxine (LT4) replacement is recommended to maintain the serum concentration of thyroid stimulating hormone (TSH) at < 2.5 μIU/ml (1st trimester) and later at < 3 μIU/ml (2nd, 3rd trimester) in women with hypothyroidism during pregnancy [5]. The clinical practice guidelines of the Endocrine Society recommend starting LT4 dosage at 50 μg/day or more in pregnant women with hypothyroidism if the serum TSH level is in a range of 2.5–10 μIU/ml [6]. However, not all pregnant women with hypothyroidism are required to increase LT4 dosage to maintain the serum level of TSH [4, 7, 8], and wide inter-individual differences in regard to increased LT4 dosage have been reported [5]. Although serum TSH level prior to pregnancy is known to be a predictor of the requirement for increasing LT4 dosage [9], there are no other known predictors of that in women with primary hypothyroidism.

Importantly, pregnant women with hypothyroidism acquired due to radioablation or surgery require a greater increase in LT4 dosage as compared to those with primary hypothyroidism [10], indicating that thyroid function reserve is closely associated with an increase in LT4 dosage during pregnancy. However, the clinical significance of inferior thyroid artery peak systolic velocity (ITA-PSV) has not been shown in patients with euthyroid Hashimoto’s disease, though we previously reported that ITA-PSV reflects thyroid gland capacity to produce thyroid hormone in not only untreated Graves’ disease [11] but also euthyroid Graves’ disease [12, 13]. Based on these findings, we postulated that ITA-PSV might reflect thyroid function reserve in patients with Hashimoto’s thyroiditis. Here, we examined the relationship of ITA-PSV with an increase in LT4 dosage in women with Hashimoto’s thyroiditis during pregnancy.

## Methods

### Study design and participants

This was a retrospective longitudinal observational study. Among female patients with Hashimoto’s thyroiditis who visited the Outpatient Clinic of the Department of Endocrinology, Osaka City University Hospital, for examination and treatment of that disease from December 2012 to January 2015, those planning pregnancy or at an early stage of pregnancy were initially considered eligible to participate in the present study (*n* = 59). Serum levels of free thyroxine (F-T4), free triiodothyronine (F-T3), and TSH were assayed using an ELECSYS™ system (Roche Diagnostics K.K., Tokyo, Japan), while ITA-PSV and thyroid volume were assessed by use of an ultrasonography method, as described later. Hashimoto’s thyroiditis was diagnosed based on a combination of clinical features, including diffuse swelling of the thyroid gland, presence of anti-thyroid microsomal or anti-thyroid peroxidase and anti-thyroglobulin antibodies, primary hypothyroidism without another cause to induce hypothyroidism, and appearance of hypoechoic and/or inhomogeneous pattern on thyroid sonogram images [14]. Those who did not become pregnant, transferred to another hospital, or had a miscarriage were excluded (*n* = 37). As a result, 22 women with Hashimoto’s thyroiditis who gave birth were retrospectively analyzed in the present study. The subjects were informed verbally regarding the study protocol, and notified that their medical records and charts may be used for research purposes. Additionally, those details as well as the opt-out method were explained in instructions posted on the hospital website (http://www.med.osaka-cu.ac.jp/interm2/research/optout.shtml). The study protocol and opt-out option were approved by the Ethics Committee of Osaka City University Graduate School of Medicine (approval No. 4070). Following approval of the study protocol, as well as receipt of administrative permission to access the medical records and charts, all data subjected to analysis were collected from relevant patient medical records and charts, and samples examined were obtained as part of routine care. The present study was conducted in full accordance with the Declaration of Helsinki.

### Ultrasound measurements of blood flow in inferior thyroid artery and thyroid volume

Using a Hitachi HI VISION Ascendus scanner (Hitachi Medical Corporation, Tokyo, Japan), ITA-PSV and thyroid volume were determined with an EUP-L75 probe operating at 18–5 MHz in both color Doppler and pulsed Doppler modes. Thyroid blood flow at the ITA was measured, as we have previously described [11–13]. Measurements were made at the ITA because this artery contributes strongly to thyroid blood flow, and measurement at this position is straightforward and has a coefficient of variation of less than 5.0%. Briefly, the angle-correction cursor was positioned parallel to the direction of flow. PSV in the right ITA was automatically calculated with the ultrasound apparatus and used as an index of thyroid blood flow. The period of training for performing ITA-PSV measurements is very short, an average of 1 week, and the clinical procedure requires only a few minutes. All measurements were performed by the same examiner (YA), who was blinded to the individual characteristics of the subjects. The intra- and interobserver coefficient of variation values were less than 5.0 and 3.0%, respectively. Thyroid volume was determined by ultrasonography, based on calculation with an ellipsoid model (width x length x thickness × 0.7, for each lobe) [15].

### Treatment of hypothyroidism with oral LT4

Serum levels of F-T4, F-T3, and TSH were routinely checked every 4 weeks during pregnancy. LT4 dosage were adjusted to maintain serum TSH at < 2.5 μIU/ml (1st trimester) and later at < 3 μIU/ml (2nd, 3rd trimester), according to the guidelines of the American Thyroid Association [5]. The increase in LT4 dosage was calculated as follows: LT4 dosage at birth - LT4 dosage at baseline.

### Statistical analysis

Values are expressed as median (interquartile range) or number (%). Spearman’s correlation coefficient test was performed to determine correlations between continuous variables. Multivariable regression analyses were performed to assess the independent influence of clinical parameters on increase in LT4 dosage. The clinical practice guidelines of the Endocrine Society recommend starting LT4 dosage at 50 μg/day or more in pregnant women with hypothyroidism if the serum TSH level is within a range of 2.5–10 μIU/ml [6], thus simple logistic regression analysis was performed to determine the associations of clinical parameters with an increase in LT4 ≥ 50 μg. In addition, analyses of the area under the curve (AUC) of receiver operating characteristics (ROC) and integrated discrimination improvement (IDI) were performed to compare the accuracy of prediction of TSH as compared to both TSH and ITA-PSV for an increase in LT4 ≥ 50 μg. Furthermore, a bootstrap optimism-corrected AUC of predictive performance was calculated for internal validation. Statistical analyses were performed using the Statistical Package for the Social Sciences software, version 18.0 (PASW Statistics version 18.0) and R, version 3.3.4 (R Foundation for Statistical Computing, Vienna, Austria). All reported *p* values are 2-tailed and were considered to be statistically significant at a value < 0.05.

## Results

### Clinical characteristics of subjects

Patient characteristics are shown in Table 1. Of the 22 enrolled, 7 (31.8%) were being administered LT4 at the beginning of study. The median levels of F-T4, F-T3, and TSH were 1.29 ng/mL, 2.97 pg/mL, and 1.71 μIU/ml, respectively. Twenty (90.9%) patients were euthyroid and 2 (9.1%) were subclinical hypothyroid status, based on elevation of serum TSH to above the normal upper limit along with a normal serum level of F-T4 [16]. The median values for ITA-PSV and thyroid volume were 14.8 cm/s and 9.2 ml, respectively.

**Table 1:** Clinical characteristics of patients

No.	22
Age, years	34.5 (33–37)
BMI, kg/m^2^	20.9 (19.3–22.9)
F-T4, ng/ml	1.29 (1.18–1.39)
F-T3, pg/ml	2.97 (2.63–3.08)
TSH, μIU/ml	1.71 (1.24–3.88)
Thyroid volume, ml	9.2 (7.3–13.1)
ITA-PSV, cm/s	14.8 (11.4–17.7)
Baseline LT4 administration, n (%)	7 (31.8)

Values are expressed as median (interquartile range) or number (%)

Abbreviations: *BMI* Body mass index, *F-T4* Free thyroxine, *F-T3* Free triiodothyronine, *TSH* Thyroid stimulating hormone, *ITA-PSV* Inferior thyroid artery peak systolic velocity, *LT4* Levothyroxine

### Association of ITA-PSV with increase in LT4 independent of serum TSH level

During pregnancy, 18 (81.8%) of the patients required an increase in LT4 dosage (12.5–100 μg). To determine whether clinical parameters including ITA-PSV had a relationship with increased LT4 dosage independent of serum TSH, multivariable regression analyses were performed (Table 2). In model 1, which simultaneously included serum TSH and ITA-PSV, both TSH and ITA-PSV were significantly and independently associated with increased LT4 dosage. Furthermore, when ITA-PSV was replaced with F-T4, F-T3, presence of LT4 administration, or thyroid volume (model 2–5), TSH alone remained significantly and independently associated with an increase in LT4.

**Table 2:** ITA-PSV and TSH independently associated with increase in LT4

	Model 1		Model 2		Model 3		Model 4		Model 5	
Variables	β	*p*	β	*p*	β	*p*	β	*p*	β	*p*
TSH	0.507	0.008	0.652	0.001	0.647	0.001	0.646	0.002	0.526	0.010
ITA-PSV	− 0.362	0.047								
F-T4			−0.194	0.268						
F-T3					−0.187	0.287				
Baseline LT4 administration (yes = 1, no = 0)							0.100	0.573		
Thyroid volume									−0.277	0.146
adjusted r^2^/p	0.475/0.001		0.392/0.003		0.389/0.004		0.361/0.005		0.420/0.002	

Standardized partial regression coefficient values (β values) and level of significance are shown

R^2^: coefficient of determination

Abbreviations: *TSH* Thyroid stimulating hormone, *ITA-PSV* Inferior thyroid artery peak systolic velocity, *F-T4* Free thyroxine, *F-T3* Free triiodothyronine, *LT4* Levothyroxine

### Associations of ITA-PSV and TSH with increase in LT4 ≥ 50 μg

During pregnancy, 7 (31.8%) patients required a dosage increase of ≥50 μg. Table 3 shows the results of simple logistic regression analysis used to examine associations between clinical parameters and an increase in LT4 ≥ 50 μg. ITA-PSV showed a significant association, while TSH had a tendency to be associated with that increase. In contrast, age, BMI, serum F-T4 and F-T3 levels, thyroid volume, and presence of baseline LT4 administration were not significantly associated with an increase in LT4 ≥ 50 μg.

**Table 3:** Simple logistic regression analysis of factors associated with increase in LT4 ≥ 50 μg

	OR (95% CI)	*p*
Age, years	1.123 (0.877–1.437)	0.359
BMI, kg/m^2^	0.924 (0.636–1.344)	0.680
F-T4, ng/ml	0.191 (0.001–56.858)	0.569
F-T3, pg/ml	0.387 (0.033–4.502)	0.448
TSH, μIU/ml	1.800 (0.972–3.336)	0.062
Thyroid volume, ml	0.812 (0.604–1.091)	0.166
ITA-PSV, cm/s	0.571 (0.348–0.935)	0.026
Baseline LT4 administration (yes = 1, no = 0)	2.062 (0.313–13.574)	0.451

Abbreviations: *OR* Odds ratio, *CI* Confidence interval, *BMI* Body mass index, *F-T4* Free thyroxine, *F-T3* Free triiodothyronine, *TSH* Thyroid stimulating hormone, *ITA-PSV* Inferior thyroid artery peak systolic velocity, *LT4* Levothyroxine

### Accuracy of TSH alone compared to that with ITA-PSV to predict LT4 increase ≥50 μg

**Fig. 1:**
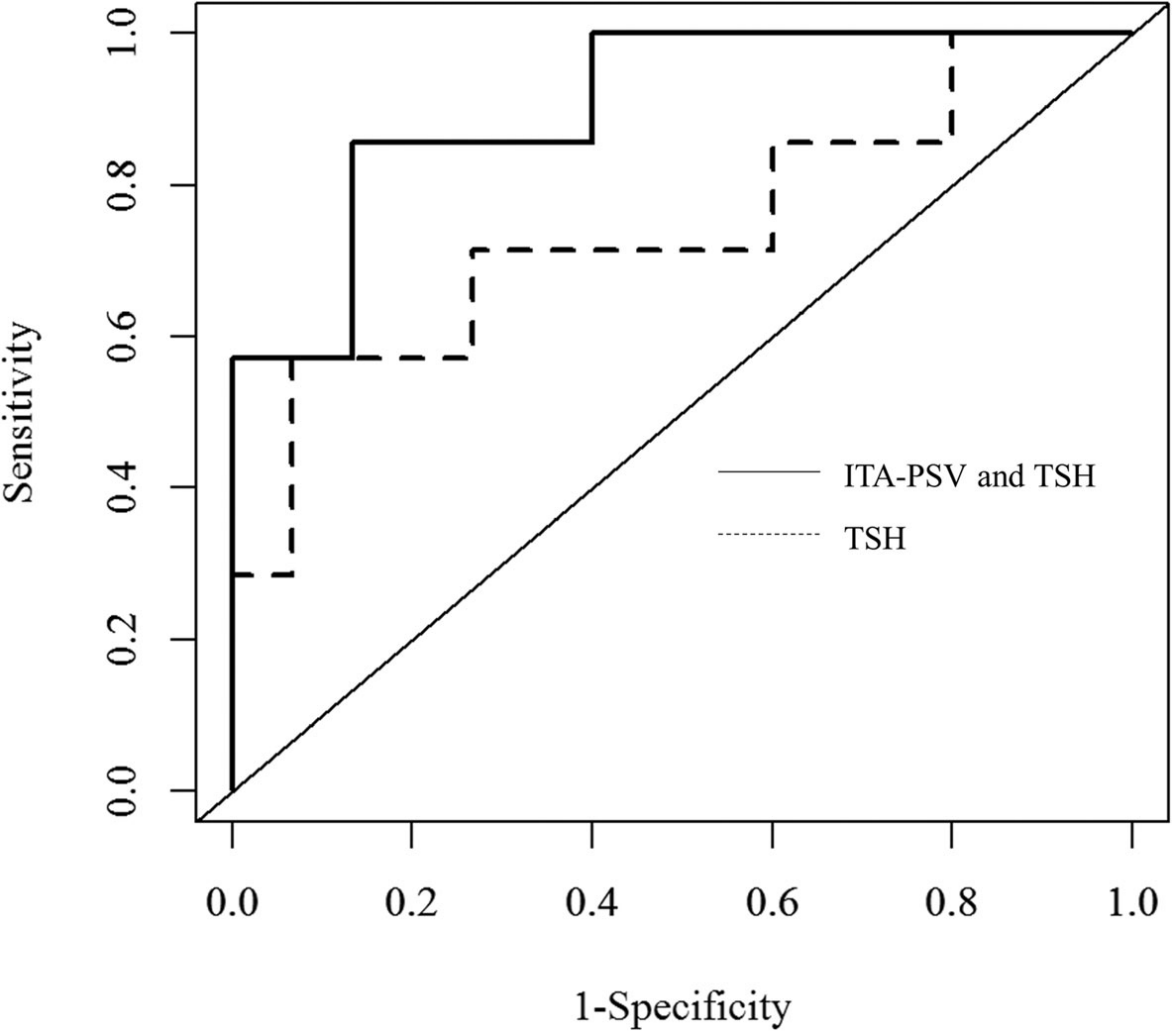
Receiver operating characteristic analysis of TSH alone as compared to ITA-PSV and TSH to predict increase in LT4 ≥ 50 μg. The AUC (0.905) value of ITA-PSV and TSH for predicting an increase in LT4 ≥ 50 μg was higher as compared to that of TSH alone (0.743), though the difference was not significant (*p* = 0.123). Abbreviations: AUC, area under the curve; TSH, thyroid stimulating hormone; ITA-PSV, inferior thyroid artery peak systolic velocity; LT4, levothyroxine

**Fig. 2:**
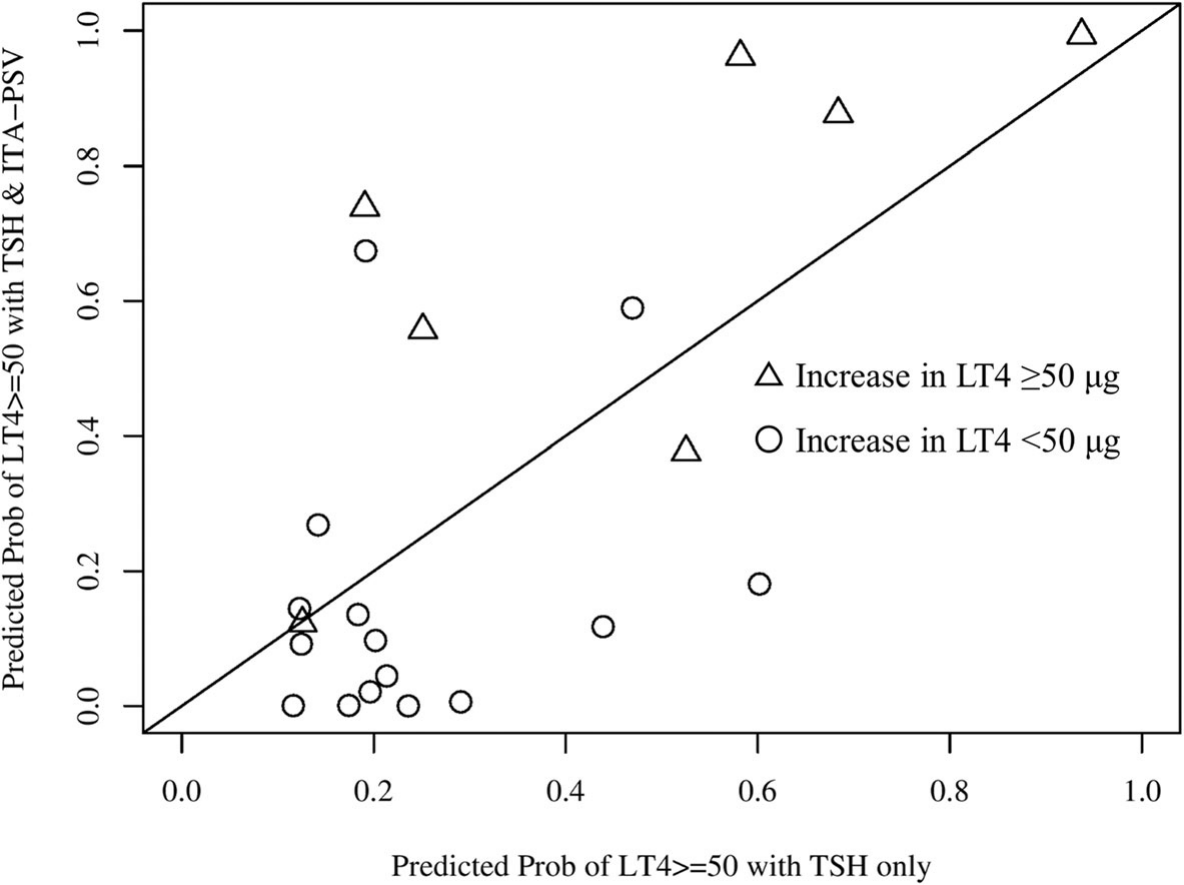
Integrated discrimination improvement analysis of TSH alone as compared to ITA-PSV and TSH to predict increase in LT4 ≥ 50 μg. The predictability of an increase in LT4 ≥ 50 μg based on both ITA-PSV and TSH was significantly increased (27.9%, *p* = 0.009) as compared to TSH alone. Abbreviations: TSH, thyroid stimulating hormone; ITA-PSV, inferior thyroid artery peak systolic velocity; LT4, levothyroxine

Serum TSH levels are routinely measured in clinical settings in patients with Hashimoto’s thyroiditis, thus we compared the accuracy of serum TSH alone to that combined with ITA-PSV for prediction of an LT4 dose increase ≥50 μg during pregnancy using ROC analysis. As shown in Fig. 1, the AUC of ITA-PSV in combination with TSH [0.905, 95% confidence interval (CI), 0.771–1.000] for predicting an increase in LT4 dosage ≥50 μg was higher as compared to that of TSH alone (0.743, 95% CI, 0.485–1.000), though the difference was not significant (*p* = 0.123). Bootstrap optimism-corrected AUC values for ITA-PSV with TSH (0.903, 95%CI, 0.810–1.000) as well as TSH alone (0.745, 95%CI, 0.505–1.000) were similar to the above findings. Furthermore, IDI (0.279, 95% CI, 0.068–0.489, *p* = 0.009) showed a significant level of discrimination improvement when ITA-PSV was added to the model (Fig. 2).

## Discussion

The present findings demonstrated that ITA-PSV is significantly associated with an increase in LT4 dosage during pregnancy independent of serum TSH, while FT4, FT3, baseline LT4 administration, and thyroid volume showed no such association. Furthermore, the combination of ITA-PSV with TSH predicted an LT4 dosage increase ≥50 μg during pregnancy more precisely than TSH alone in pregnant women with Hashimoto’s thyroiditis. Together, our results suggest that ITA-PSV independent of TSH is useful to reflect thyroid function reserve to increase thyroid hormone production during pregnancy.

Previous investigations have shown that 50 to 85% of women with hypothyroidism require an increase in LT4 dosage during pregnancy [4, 7, 8], though there are wide inter-individual differences in regard to such an increase given to compensate for the increased demand for thyroid hormone production while pregnant [5]. Consistent with those reports, 81.8% of the present pregnant patients required an LT4 dosage increase for maintaining serum TSH level within the target range, with that increase level varying from 12.5 to 100 μg in the present study. Abalovich M et al. reported that a higher TSH level prior to pregnancy is predictive of a requirement for increased LT4 dose during pregnancy in women with primary hypothyroidism, in whom serum TSH prior to pregnancy was < 2.5 μIU/ml [9], suggesting that serum TSH level might reflect the current status of thyroid hormone production even in women with euthyroid Hashimoto’s thyroiditis. In agreement with that report, the present study found that a high serum level of TSH was independently associated with an increase in LT4 dose and also had a tendency to be associated with an increase in dosage ≥50 μg. However, pregnant women with hypothyroidism due to undergoing radioablation therapy or a total thyroidectomy were previously reported to require a greater increase in LT4 dosage during pregnancy as compared to those with primary hypothyroidism, even when baseline TSH normalized with LT4 replacement therapy did not differ significantly prior to pregnancy between those two patient groups [10]. Those findings reflected a lack of residual thyroid function to increase hormone production in response to an increased hormone demand during pregnancy in the former group of patients in that study.

Although thyroid blood flow was shown to be increased in patients with clinically overt hypothyroidism as compared to healthy individuals [17], that level was reported to be lower as compared to patients with a TSH-producing tumor [18], suggesting that a rise in ITA-PSV in response to an increase in TSH is weak in patients with Hashimoto’s thyroiditis due to destruction of the thyroid gland. Importantly, we previously showed that ITA-PSV predicts Graves’ disease relapse after anti-thyroid drug (ATD) withdrawal [12] as well as after normal delivery in patients with euthyroid Graves’ disease [13], while a maintenance dosage of ATD is required to keep serum TSH level within a normal range in those patients who are otherwise untreated [11]. Interestingly, in patients with euthyroid Graves’ disease, ITA-PSV was shown to be correlated with serum VEGF level, known to be a marker of intra-thyroid angiogenesis, whereas neither serum F-T4 nor F-T3 was found to have a relationship [12], suggesting that ITA-PSV reflects thyroid hormone production in euthyroid Graves’ disease. In contrast, we previously reported that ITA-PSV was not correlated with the level of F-T4 or F-T3 in healthy subjects [11]. In addition, though ITA-PSV was not significantly correlated with serum F-T4 (ρ = 0.183, *p* = 0.414), F-T3 (ρ = 0.091, *p* = 0.687), or TSH (ρ = − 0.262, *p* = 0.239) in the present study, it was independently associated with an increase in LT4 dosage, suggesting that ITA-PSV reflects residual thyroid capability to increase thyroid hormone production, but not current thyroid hormone production capability, in patients with euthyroid or subclinical Hashimoto’s thyroiditis.

This study has some important limitations. First, it was retrospective in design. Second, the patient cohort was nearly exclusively Japanese individuals, thus it is unclear whether these findings can be generalized for other ethnic groups. Third, the number of subjects investigated was low. Therefore, the power of the present findings is low, and we were not able to fully estimate the association of gestational age with evaluation or sensitivity of ITA-PSV. Our findings should be confirmed in a large-scale study. Nevertheless, to the best of our knowledge, no previously reported study has performed such an examination of predictors of increased LT4 outside of TSH level.

## Conclusions

In pregnant women with Hashimoto’s thyroiditis, ITA-PSV and TSH levels were independently associated with a required increase in LT4 dosage, while those together more precisely predicted that requirement.

## Data Availability

The datasets used and/or analyzed during the current study are available from the corresponding author on reasonable request.

## Abbreviations

ATD: anti-thyroid drug
AUC: area under the curve
CI: confidence interval
F-T3: free triiodothyronine
F-T4: free thyroxine
IDI: integrated discrimination improvement
ITA-PSV: inferior thyroid artery peak systolic velocity
LT4: levothyroxine
ROC: receiver operating characteristics
TSH: thyroid stimulating hormone
